# Tuning the electrical conductance of metalloporphyrin supramolecular wires

**DOI:** 10.1038/srep37352

**Published:** 2016-11-21

**Authors:** Mohammed Noori, Albert C. Aragonès, Giuseppe Di Palma, Nadim Darwish, Steven W. D. Bailey, Qusiy Al-Galiby, Iain Grace, David B. Amabilino, Arántzazu González-Campo, Ismael Díez-Pérez, Colin J. Lambert

**Affiliations:** 1 Department of Physics, Lancaster University, Lancaster, LA1 4YB, UK; 2 Department of Physics, Collage of Science, Thi-Qar University, Iraq; 3 Department of Physical Chemistry, University of Barcelona, Diagonal 645, Spain; 4 Institute for Bioengineering of Catalonia (IBEC) Baldiri Reixac 15-21, 08028 Barcelona, Catalonia, Spain; 5 Centro Investigación Biomédica en Red (CIBER-BBN). Campus Río Ebro-Edificio I+D, Poeta Mariano Esquillor s/n, 50018 Zaragoza, Spain; 6 Institut de Ciència de Materials de Barcelona (ICMAB-CSIC), Campus Universitari, 08193 Bellaterra, Catalonia, Spain; 7 Physics Department, College of Education, Al-Qadisiyah University, Iraq; 8 School of Chemistry, The University of Nottingham, University Park, Nottingham, NG7 2RD, UK

## Abstract

In contrast with conventional single-molecule junctions, in which the current flows parallel to the long axis or plane of a molecule, we investigate the transport properties of M(II)-5,15-diphenylporphyrin (M-DPP) single-molecule junctions (M=Co, Ni, Cu, or Zn divalent metal ions), in which the current flows perpendicular to the plane of the porphyrin. Novel STM-based conductance measurements combined with quantum transport calculations demonstrate that current-perpendicular-to-the-plane (CPP) junctions have three-orders-of-magnitude higher electrical conductances than their current-in-plane (CIP) counterparts, ranging from 2.10^−2^ G_0_ for Ni-DPP up to 8.10^−2^ G_0_ for Zn-DPP. The metal ion in the center of the DPP skeletons is strongly coordinated with the nitrogens of the pyridyl coated electrodes, with a binding energy that is sensitive to the choice of metal ion. We find that the binding energies of Zn-DPP and Co-DPP are significantly higher than those of Ni-DPP and Cu-DPP. Therefore when combined with its higher conductance, we identify Zn-DPP as the favoured candidate for high-conductance CPP single-molecule devices.

Porphyrins offer a variety of desirable features as building blocks for future molecular-scale devices including their highly-conjugated structure, rigid planar geometry, high chemical stability and their ability to form metalloporphyrins by coordinating metal ions in the center of their macrocyclic and aromatic skeleton^1^,^2^,^3^,^4^,^5^. Following early work, which established their chemical and biological properties^6^,^7^,^8^,^9^, porphyrins have become a focus of interest both for experimental and theoretical investigations of molecular electronics^10^,^11^,^12^ and for the design of complexes using supramolecular chemistry, leading to a diverse array of structures available for nano-scale building blocks^13^. This unique combination of properties is exploited in nature, where for example metalloporphyrins acts as charge carriers in naturally occurring processes such as photosynthesis^14^,^15^,^16^,^17^ and in the respiratory chain^18^,^19^. In many of these processes, the plane of the porphyrin skeleton is stacked perpendicular to the direction of charge transport, whereas previous studies^10^,^11^,^12^ address conductance with the plane of the porphyrin skeleton aligned parallel to the direction of charge transport. In the latter “current in plane” (CIP) up-right configuration (Fig. 1a), the porphyrin skeleton was contacted to gold electrodes via thiol or pyridyl anchor groups and the electrical conductance was found to be low^10^,^20^ (of order nanosiemens). For the purpose of developing future single-molecule electronics and thermoelectrics, it is highly desirable to increase the electrical conductance, since this can lead to higher switching speeds and reduce the relative effect of parasitic phonons in thermoelectric devices. In what follows we develop a strategy for increasing the electrical conductance of porphyrin-based single-molecule wires by investigating their conductance with the current perpendicular to the plane (CPP) (Fig. 1b). We report a joint experimental and theoretical study of CPP conductance trends and binding configurations across a family of 5,15-diphenylporphyrins (DPPs), with a centrally-coordinated divalent metal ion of either Co(II), Ni, Cu or Zn and demonstrate that their conductance and stability can be tuned through the choice of metal atom. This is an extension of previous experimental measurement^21^ which showed that the CPP conductance of the flat-laying sandwiches of a Co(II)-DPP shows a large conductance value of three orders of magnitude higher than the measured in-plane conductance^10^.

## Results and Discussion

### Binding energies and relaxed configurations

To obtain theoretical results for binding energies and relaxed configurations, spin-polarised DFT calculations were carried out using SIESTA^22^ with the local density functional approximation parameterised by Ceperley and Adler^23^. Initially the geometry of each isolated porphyrin was optimised to a force tolerance less than 20 meV/Å using an extended double zeta polarised basis set of pseudo atomic orbitals for all atoms, and a mesh cutoff of 200 Ry to define the real space grid. Next, the binding energy *E*^*B*^of a single pyridine-4-yl-methanol (PY) with the porphyrin was calculated using the counterpoise method^24^,^25^ (see SI). For all four metallo-porphyrins, we find that the energetically-most-favorable configuration occurs when the PY nitrogen atoms are located above the metal atom of the porphyrin. For this most-favorable position of the PY nitrogen atoms, the results for all four binding energies and the corresponding nitrogen-metal distances are shown in Table 1.

### Conductance measurements

The Co-DPP and Zn-DPP molecules were synthesized according to the procedure described by Song *et al*.^26^. The synthesis of Cu-DPP and Ni-DPP described by^26^ was modified by changing the solvent and reaction times (See SI for experimental details). PY was synthesized as described previously by Puigmarti-Luis *et al*.^27^ and the single-molecule transport measurements were conducted following the procedure described in ref. 21.

**Figure 2:**
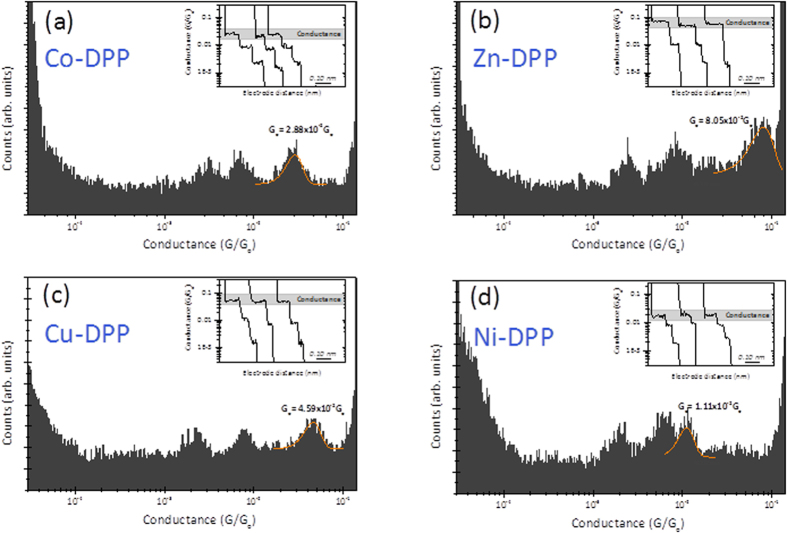
(**a,b,c and d**) show the semi-log conductance histograms for the experimental STM single-molecule transport experiment for the Co-DPP, Zn-DPP, Cu-DPP and Ni-DPP systems, respectively. The inset shows representative single current decay curves used to build the conductance histograms. The applied BIAS was set to +25 mV. The sharp increase in counts in both left and right sides of the histograms correspond to the current amplifier baseline and saturation respectively.

Briefly, an Au(111) surface and a STM Au-electrode tip were both functionalized with ∼1 mM PY solution in ethanol and incubated for 24 h (See Experiments SI for further details). Both electrodes were mounted onto the STM cell and the cell was filled with mesitylene, an inert, non-polar organic solvent, in which the target M(II)-porphyrin is solubilized in nano-molar concentrations^25^. Details about the STM-break junction (STM-BJ) measurements can be found elsewhere (see SI). Briefly, the STM-BJ experiments consist of repeatedly approaching and retracting the two pyridyl-functionalized electrodes, while monitoring the tunneling current flowing through the electrode-electrode STM junction under a low applied voltage bias (±10 and ±25 mV). ~5000 current traces were collected and 10–15% of them were used to build a conductance 1D histogram for each molecule, examples of which are shown in Fig. 2 insets. As control experiments, single-molecule conductance experiments in the absence of porphyrin molecules and in the presence of empty DPP were performed under the same experimental conditions (see details in Supporting Information). The absence of porphyrin molecules resulted in the lack of molecular junction events and the DPP showed no high conductance peak in the histograms. The fit of the observed high conductance peak in Fig. 2 histogram was used to extract a most-probable value of the single-molecule conductance for the flat-stacked metalloporphyrin^21^. The observed two low conductance peaks are commonly-observed for all porphyrins and they have been ascribed to molecular wires with more extended (tilted) conformations of the porphyrin bridging the gap at longer electrode-electrode separations^25^. The fact that the empty DDP uniquely displays the low conductance features is evidence that such conformations arise from the interaction between the PY and the porphyrin ring moieties. The conductance values extracted from Gaussian fits to the conductance histograms for each metalloporphyrin (Fig. 2) has been also supported by a static *blinking* STM approach, where the spontaneous formation of the porphyrin bridge is attained while holding a fixed electrode-electrode distance (see SI for more details on the *blinking* method).

### Conductance calculations

**Figure 3:**
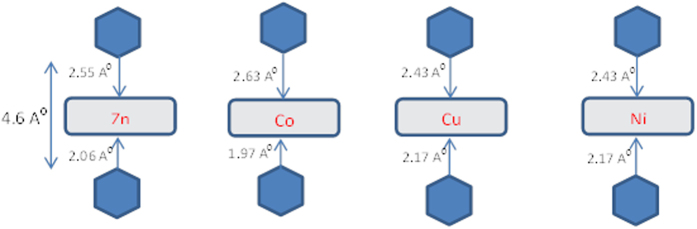
Scheme of contact of pyridine anchor above the porphyrin molecule. The lower PY nitrogen is a distance *d* from the metal atoms, while the the upper PY nitrogen is placed a distance 4.6 Å above the lower PY nitrogen.

To model an example of a blinking experiment in which the electrodes are held at a fixed separation, we fixed the PY-functionalised gold electrodes at separation corresponding to a 4.6 Å distance between the terminal N atoms of the PYs, as shown in Fig. 3. This distance is chosen to be slightly larger than the highest value of the distances *d* in table 1, such that all molecules can be accommodated within the electrode gap. We then allowed the porphyrin molecule to bind to the lower PY, with a N-to-metal-atom distance of *d* (see Table 1). The PY of the upper gold electrode was therefore more weakly bound to the metal atom of the porphyrin, as would be the case in a blinking experiment.

Before computing transport properties, we first examined the spin state of the metalloporphyrins. Numerous studies have examined the effect of the axial ligand on the redox^28^,^29^ and photovoltaic properties of metalloporphyrins^30^. Nickel porphyrin with coordinating axial ligands are paramaganetic (S = 1) in contrast to four-coordinate species (S = 0)^31^,^32^. Therefore, to accurately calculate the transport properties of these molecules spin polarized transport calculations must be carried out. We find in the case of the zinc-metalloporphyrin were there is no spin dependence the up spin and down spin transmission curves are almost identical (See Fig. S1).

The conductance was then calculated using the Gollum quantum transport code^33^, which utilizes the mean-field Hamiltonians provided by DFT. Starting from the SIESTA Hamiltonian, we use Gollum to calculate the transmission coefficient *T*^*σ*^(*E*), describing electrons of energy *E*, spin *σ* = [↑, ↓] passing from one electrode to the other via the porphyrin, from which the finite-temperature electrical conductance *G* is obtained using the Landauer formula


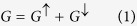



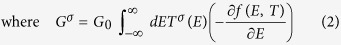


In this expression, *f*(*E*, *T*) is the Fermi distribution function defined as 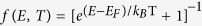 where *k*_*B*_ is Boltzmann’s constant and 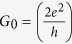 is the quantum of conductance.

**Figure 4:**
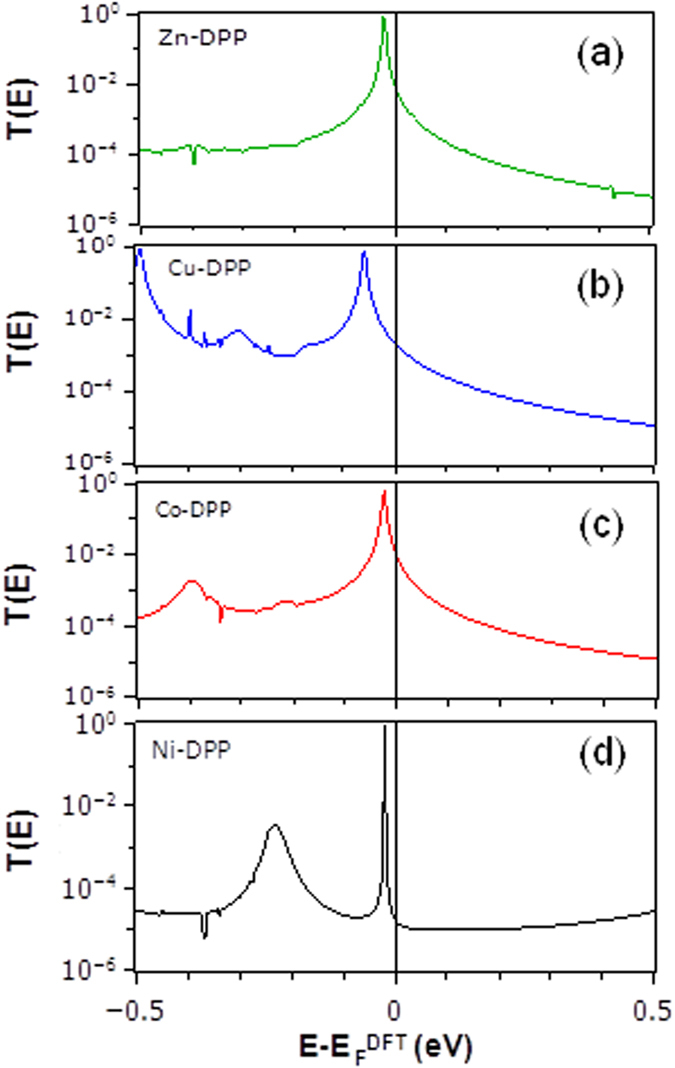
The total transmission coefficient as a function of energy for (**a**) Zn-DPP, (**b**) Cu-DDP, (**c**) Co-DPP and (**d**) Ni-DDP. Each PY-porphyrin is in its relaxed configuration, with the metal atom a distance *d* from the N of the lower PY. The upper PY-functionalised gold electrode was then positioned such that distance between the upper and lower PY nitrogens was fixed at 4.6 Å.

**Figure 5:**
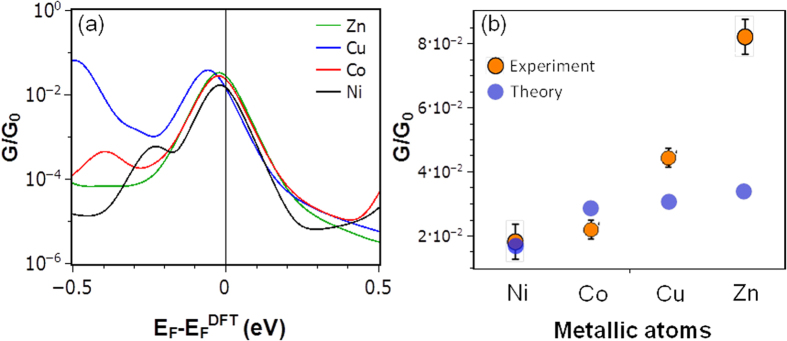
The calculated room-temperature electrical conductances for Zn-DPP, Co-DPP, Cu-DPP and Ni-DPP, obtained from Fig. 4. (**b**) Comparison between experimental (orange circles) and theoretical conductances (blue circles) obtained by choosing an optimum values of E_F_ − E_F_^DFT^ = −0.03 eV. The error bars in the experimental points (orange circles) represent the full width at half maximum from the corresponding conductance histogram peak in Fig. 2, which were obtained from the accumulation of hundreds of individual traces for every system.

Figure 4 shows the total transmission coefficients as a function of energy for Zn-DPP, Cu-DPP, Co-DPP and Ni-DPP respectively. The corresponding room-temperature conductances versus Fermi energy *E*_*F*_ are shown in Fig. 5a. Since the Fermi energy 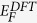
predicted by DFT is not necessarily accurate^34^, to compare theory with experiment, we treat the Fermi energy *E*_*F*_ as a single free parameter, chosen to determine four conductances, which are closest to the experimental trend. Figure 5b shows that the experimentally-measured order Ni < Co < Cu < Zn is obtained by choosing a Fermi energy 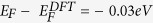.

Figure 5b shows that the chosen junction separation captures the experimental ordering of the Ni-DPP, Co-DPP, Cu-DPP and Zn-DPP. Furthermore, the computed magnitudes of the conductances are of the same order as the measured values and these conductances are far higher than those measured for CIP junctions, which are typically less than 10^−4^ G_0_.

## Conclusion

We have investigated the electrical conductance with the current perpendicular to the plane (CPP) of supramolecular metalloporphyrin wires. Both theory and experiment reveal that the variation in conductance across this family of molecules increases in the order Ni < Co < Cu < Zn. Experimentally the conductance of Zn-DPP is found to be a factor of 4 greater than that of Ni-DPP. Crucially the CPP conductances are three orders of magnitude greater than their CIP counterparts. For example as reported in [10] for Zn-porphyrins, the CIP conductance is 2.7·10^−5^ G_0_, which is more than three orders of magnitude lower than our measured CPP conductance. Similarly in [2] the reported CIP conductances for Cu, Co and Ni porphyrins were 3.6 10^−5^ G_0_, 2.5 10^−5^ G_0_ and 1.9 10^−5^ G_0_ respectively. This supramolecularly-wired arrangement with the aromatic plane perpendicular to the current is therefore stable at room temperature and provides a unique family of high-conductance molecular wires, whose electrical conductances and binding energies can be tuned by metal substitution. From the point of view of stability, we find that the binding energies of Zn-DPP and Co-DPP are significantly higher than those of Ni-DPP and Cu-DPP and therefore in view of its higher conductance, we identify Zn-DPP as the favoured candidate for high-conductance CPP single-molecule devices.

## Additional Information

**How to cite this article**: Noori, M. *et al*. Tuning the electrical conductance of metalloporphyrin supramolecular wires. *Sci. Rep.* **6**, 37352; doi: 10.1038/srep37352 (2016).

**Publisher’s note:** Springer Nature remains neutral with regard to jurisdictional claims in published maps and institutional affiliations.

## Supplementary Material

Supplementary Information

## Figures and Tables

**Figure 1:**
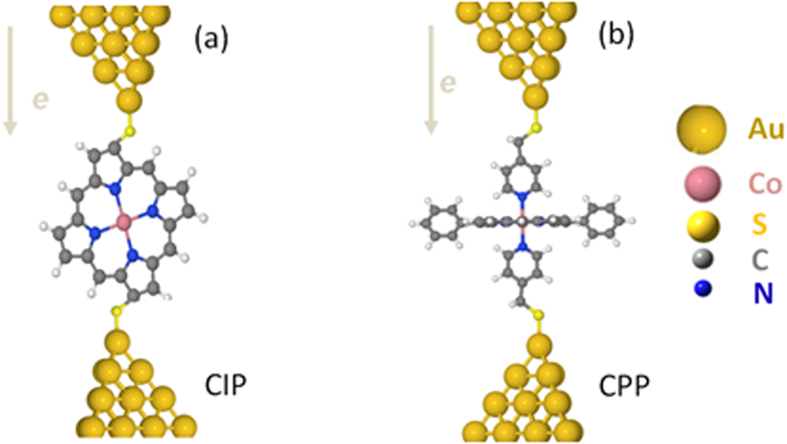
(**a**) Porphyrin skeleton aligned parallel to the direction of charge transport “current in plane” (CIP) up-right configuration and (**b**) the optimised sandwich configuration of DPP junction with the current perpendicular to the plane (CPP).

**Table 1:** Shows optimum distance (*d*), and binding energies *E* ^*B*^ for all four metalloporphyrins.

Metal	*d* Å	*E*^*B*^ eV
Zn	2.06	−1.21
Cu	2.17	−0.45
Co	1.97	−1.20
Ni	2.17	−0.17
