# Supplementary material for: Tuning the electrical conductance of metalloporphyrin supramolecular wires
Source: Sci Rep. 2016 Nov 21;6:37352. doi: 10.1038/srep37352 (PMC5116753; doi:10.1038/srep37352)
Supplement: Supplementary Information [file srep37352-s1.pdf]

Supplementary information.

Tuning the electrical conductance of metalloporphyrin supramolecular wires.

Mohammed Noori[†, &], Albert C. Aragonès[*,#], Giuseppe Di Palma[¶], Nadim Darwish[*], Steven W. D. Bailey[†], Qusiy Al-Galiby[†, $], Iain Grace[†], David B. Amabilino[‡], Arántzazu Gonzalez-Campo[¶], Isma Díez-Pérez[*,#] and Colin J. Lambert[†].

[†] Department of Physics, Lancaster University, Lancaster, LA1 4YB, UK

[*] Department of Physical Chemistry, University of Barcelona, Diagonal 645, Spain & Institute for Bioengineering of Catalonia (IBEC) Baldiri Reixac 15-21, 08028 Barcelona, Catalonia, Spain

[#] Centro Investigación Biomédica en Red (CIBER-BBN). Campus Río Ebro-Edificio I+D, Poeta Mariano Esquillor s/n, 50018 Zaragoza, Spain.

[¶] Institut de Ciència de Materials de Barcelona (ICMAB-CSIC), Campus Universitari, 08193 Bellaterra, Catalonia, Spain

[‡] School of Chemistry, The University of Nottingham, University Park, Nottingham, NG7 2RD, UK

[&] Department of Physics, Collage of Science , Thi-Qar University, Iraq.

[$] Physics Department, College of Education, Al-Qadisiyah University, Iraq.

Corresponding author: c.lambert@lancaster.ac.uk

Theory

To accurately calculate the binding energies E^B, basis set superposition errors were avoided by retaining 'ghost states' as prescribed in the counterpoise method [23,24], using the formula

$$E^B = E_{AB} - (E_{aB} + E_{Ab}) \qquad (1)$$

In this expression the total bound state energy of entity A bound to B is E_{AB} , the total energy of B in the presence of the ghost states a is E_{aB} and the total energy of A in the presence of the ghost states b is E_{Ab}.

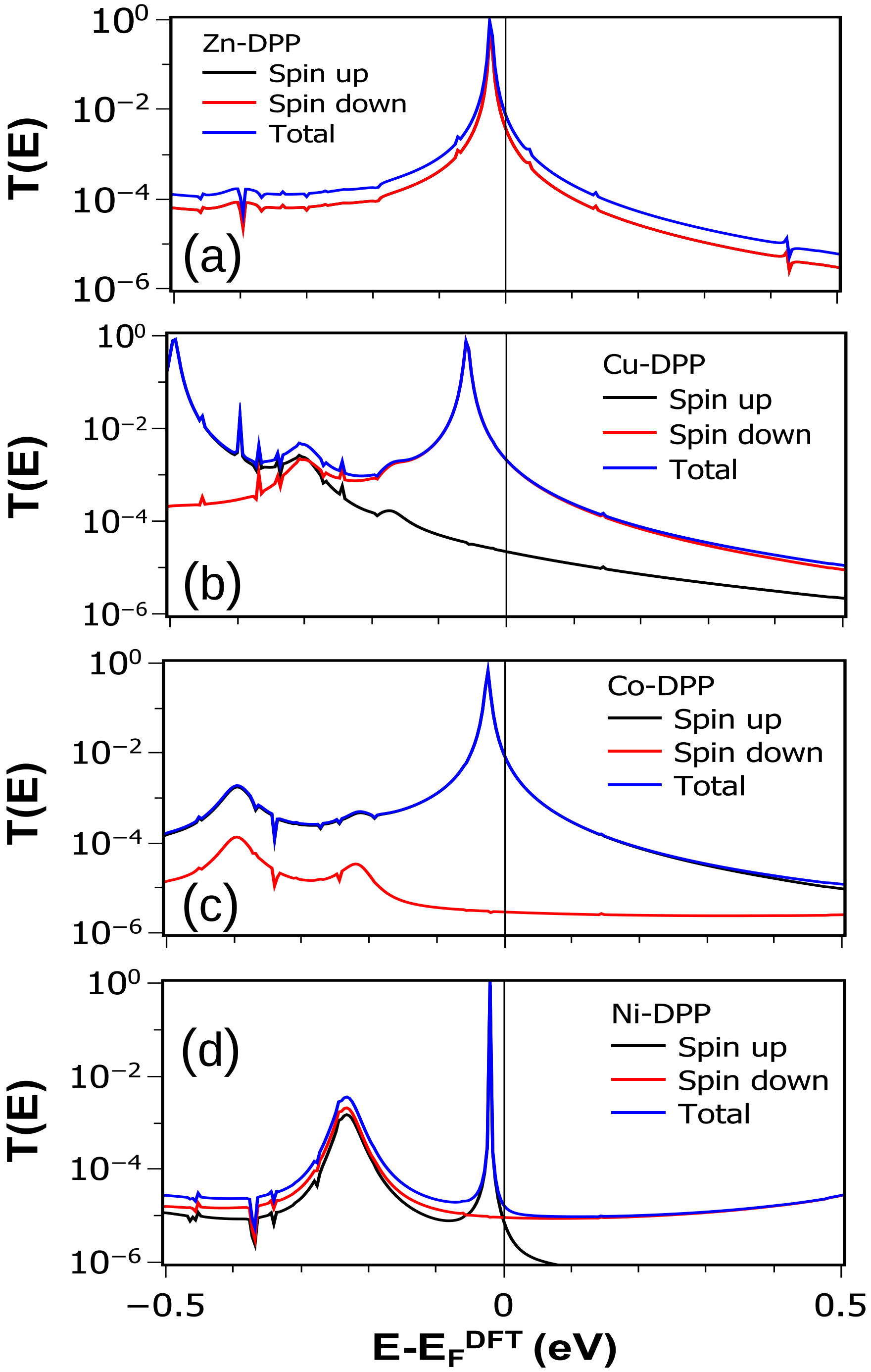

Figure S1. The spin up, spin down and total transmission coefficient as a function of energy for (a) Zn-DPP, (b) Cu-DDP, (c) Co-DPP and (d) Ni-DDP. Each PY-porphyrin is in its relaxed configuration, with the metal atom a distance d from the N of the lower PY. The upper PY-functionalised gold electrode was then positioned such that distance between the upper and lower PY nitrogens was fixed at 4.6 Å.

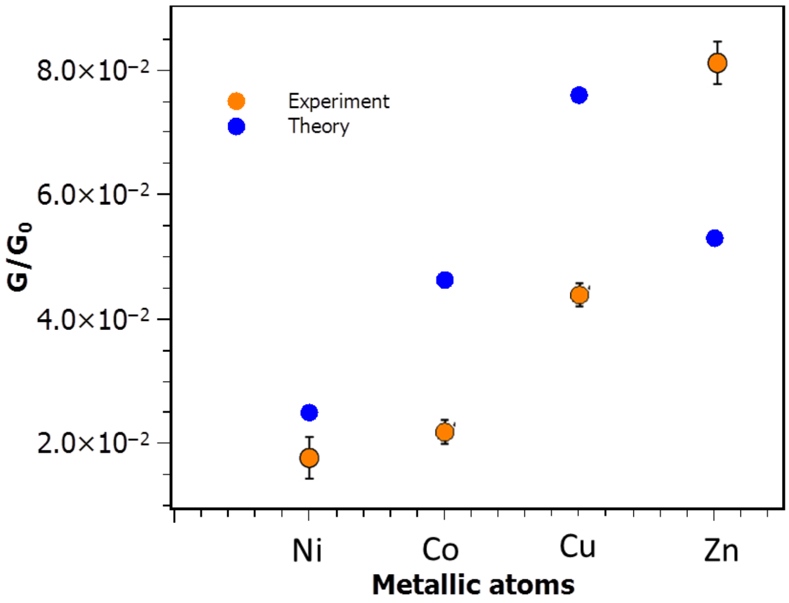

Figure S2. Comparison between experimental (orange circles) and theoretical conductances (blue circles) obtained by choosing an optimum values of E_F - E_F^{DFT} = -0.05 eV.

Experiments.

All glassware and PTFE STM-cells were cleaned with piranha solution (1:1 H2SO4/H2O2 by volume) before usage followed by rinsing with 18 MΩ cm−1 Milli-Q water (Millipore). An Au (111) single crystal substrate (10 mm x 1 mm) of 99.9999% purity and orientation accuracy < 0.1 degrees was purchased from MaTeck (Germany). Before each experiment, the single crystal Au (111) substrate was electropolished to eliminate possible residual contamination and then annealed with a H2 flame. The surfaces were then washed with argon-purged ethanol and dried under a stream of argon after which they were placed in 0.8-1 mM ethanol solution of pyridin-4-yl-methanethiol for 24 h. Au tips were mechanically cut, briefly H2 flame annealed and immediately immersed in 0.8-1 mM ethanol solution of pyridin-4-yl-methanethiol for 24 h. The Au (111) surfaces and Au tips were then washed thoroughly with ethanol and dried under a stream of argon. The Au (111) surface was then assembled in the STM cell and the surface electrode filled with an 80 µL of pure mesitylene, where STM junction control experiments were firstly run. Next, few drops of an 8-12 nM mesitylene solution of the porphyrin was added to build the porphyrin mediated molecular wires27.

Single-molecule conductance experiments in the absence of porphyrin molecules and in the presence of the empty DPP are presented in Figure S3a and b respectively. The former show no molecular junction events whereas the latter show no high conductance peak, displaying the two low conductance features associated of the interaction between PY and porphyrin backbone27.

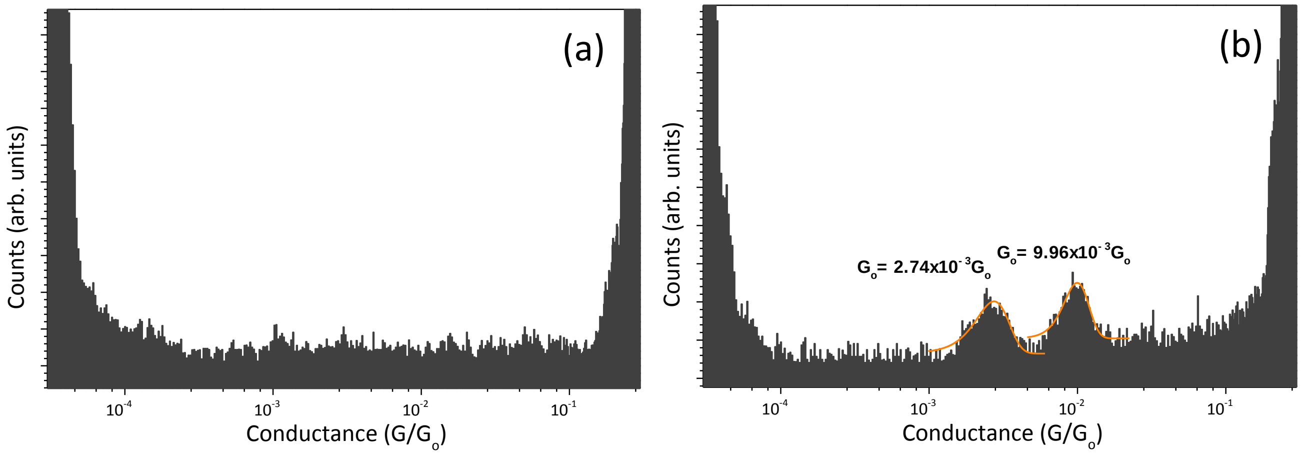

Figure S3. Semi-log conductance histograms of the experimental STM break-junction single-molecule transport experiments in the absence of porphyrin (a) and in the presence of the empty DPP (b). The applied BIAS was set to +25mV.

Materials and Methods

The 5,15-diphenylporphyrin (DPP) was purchased from Frontier Scientific. The Ni(OAc)2·4H2O and Cu(OAc)2·H2O salts were purchased from Sigma-Aldrich and used as received. The IR spectra were recorded on a Shimazu FTIR-8300 spectrophotometer. The 1H-NMR (300 MHz) spectra were recorded on a BRUKER ARX 300 spectrometer. MALDI-TOF-MS mass spectra were recorded in the positive ion mode using a Bruker Ultraflex MALDI-TOF/TOF spectrometer. UV-Vis spectra were recorded on a Varian Cary 5000 UV-Vis-NIR spectrophotometer.

Technical details of the single-molecule transport measurements

Dynamic STM-break junction approach. Details about the STM-break junction (STM-BJ) technique have been published elsewhere.S1 All the conductance measurements were carried out with a mechanically and electronically isolated PicoSPM II microscope head controlled by a Picoscan 2500 electronics (all from Agilent, USA) and using a homemade PTFE-STM cell. Data captures were acquired using a NI-DAQmx/BNC-2110 National Instruments (LabVIEW data acquisition System, USA) and analyzed with LabVIEW code. The procedure of a typical break-junction experiment is based on first bringing the STM tip to a tunneling distance over a flat Au (111) surface area. The STM feedback is then turned off and the tip is driven into and out of contact with the substrate at a speed of 1-2V/s. These 2-points feedback loop is used to collect thousands of current decays (5000-6000). Single

molecule conductance (G) was determined using the expression G=Istep/UBIAS, where I is the current and U is the voltage potential difference between the two electrodes. The current decays are accumulated to semi-logarithmic conductance histograms. The observed plateaus in the individual current decays result in the observed peaks in the conductance histograms and provide an averaged value of the single-molecule conductance. Transient curves that are either noisy or that showed smooth exponential decay due to the absence of molecular bridge formation were rejected when building the histograms using an automatic selection procedure driven by a LabVIEW code. The histograms were compiled by applying the same automated selection criteria to each set of all the recorded decay curves. The percentage of decay curves that showed plateau features were typically 10–15 % and were all selected to build the histograms. This selection process make peaks in the conductance histograms protrude above the tunneling background and allow a quantitative measure of the yield of molecular junction formation in all of the measured series.

Static blinking approach. In order to study the interaction of the PY with the metal or with the porphyrin skeleton, current transient captures were carried out at a fixed distance such that spontaneous formation of molecular junctions is attained. Briefly, imposing a set point tunneling current first sets an initial distance between the functionalized electrodes. The STM feedback is then turned off and the tunneling current is recorded as a function of time. When the molecular junction is spontaneously formed, the current abruptly increases S2, S3. The observed current "jumps" are referred to as "blinks". The blinks typically last for a short period of time, after which the current suddenly drops returning to the set point tunneling current due to the spontaneous breakdown of the molecular junction. A representative blink for the Co-DPP capture is shown in Figure S3b. Tens of the individual blinking features are set to a common time origin and background and accumulated into a single 2D blinking map (Figure S3a). The obtained average conductance value from S3a is fully consistent with the value obtained from the dynamic STM break-junction experiments. The lifetime of the blinks formed via PY interaction with Co-DPP metal center (junction lifetime from the X-axis in S3) present large values >1.5 seconds significantly larger than the low conductance features ascribed to the porphyrin ring. This fact is consistent with the lower binding specificity as compare to the high conductance feature resulting from the PY metal complexation.

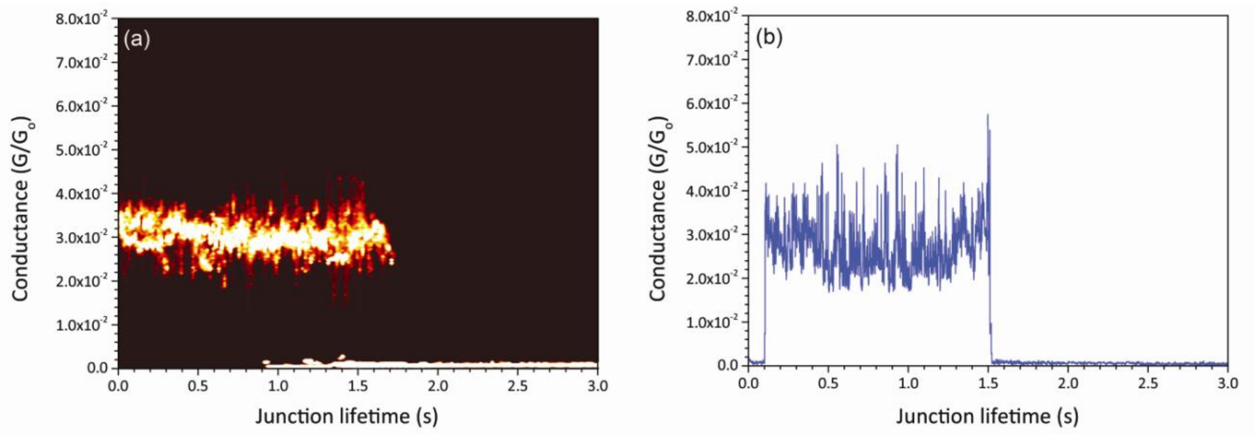

Figure S4. Representative individual "blinks" corresponding to the formation of molecular junctions with the Co-DPP molecule through the metal ion (blue trace) or with the DPP molecule through the porphyrin skeleton (grey and black traces). The applied bias was set to 10 mV for both molecules. The lifetime of the former case is significantly larger than the DPP examples (absence of metal center).

Synthesis of Ni-DPP

To a solution of 100 mg (216 mmol) of DPP in dry DMF (30 mL) was added 128 mg (707 mmol) of Ni(OAc)2·4H2O and the mixture was stirred at 120 oC for 6h and then at room temperature overnight. After this time, the solvent was removed and the residue was extracted with brine/hexane and the organic phase was dried with MgSO4. Evaporation of the volatiles at reduced pressure gave a crude product, which was crystallized with CH2Cl2 and MeOH, to obtain a red solid. Yield: 55 mg, 47 %. 1H NMR (CDCl3), δ (ppm): 9.96 (s, 2H, Hmeso), 9.21 (d, 4H, J= 6Hz, β-H), 8.96 (d, 4H, J=6Hz, β-H), 8.10 (d, 4H, J=9Hz Horto) 7.76 (m, 6H, Hmeta and Hpara). IR (νmax/cm-1): 1253, 1068, 966, 904, 853, 773, 725, 703. MALDI-TOF-MS (m/z): Calcd for C32H20N4Ni: 518.10. Found: 518.22 [M]. UV-Vis (CH2Cl2, 25oC): λmax (ε) nm 390 (13089), 394 (13089), 396 (13089), 399 (13089), 405 (13089), 516 (875), 548 (450).

Synthesis of Cu-DPP

To a solution of 40 mg (86.4 mmol) of DPP in dry DMF (10 ml) was added 48 mg (265 mmol) of Cu(OAc)2·H2O and the mixture was stirred at 120°C for 4h. After this time, the solvent was removed and the residue was extracted with CH2Cl2 and dried with MgSO4. Evaporation of the volatiles gave a crude product, wich has crystallized with CH2Cl2/MeOH obtaining a purple solid. Yield: 22 mg, 55%. IR (νmax/cm-1): 697, 723, 783, 854, 994, 1065, 2285. MALDI-TOF-MS (m/z): Calcd for C32H20N4Cu: 523.09. Found: 523.24 [M]. UV-Vis (CH2Cl2, 25oC): λ_{max} (ε) nm 403(106052), 528(138974), 562(147894).

XPS was conducted on a thoroughly washed Au(111) surface previously modified with pyridine followed by modification with Zn-DPP. The presence of pyridinyl units is confirmed by the observation of Au-S bond energy shift. The presence of Zn-DPP on the distal end of the surface is confirmed by the observation of metallic Zinc at 1021.7 eV.

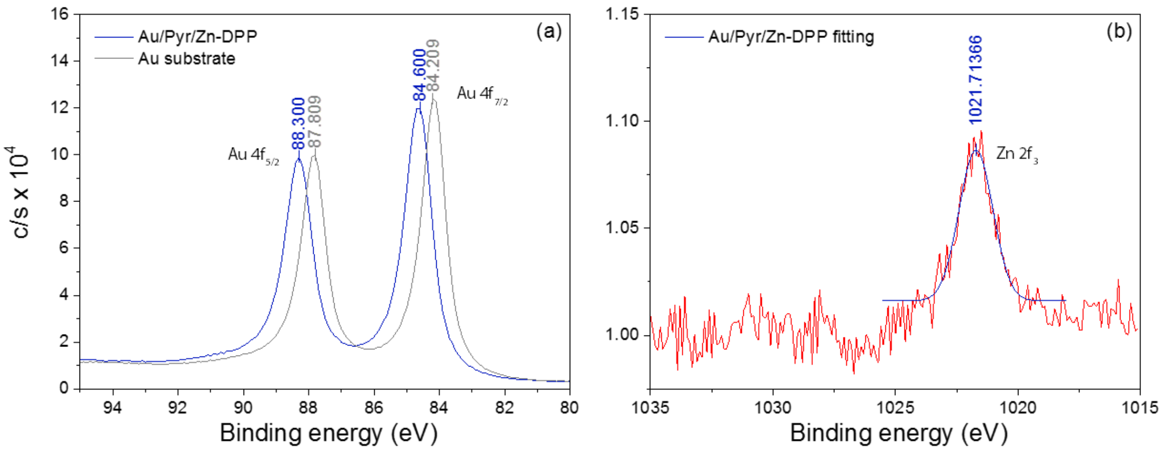

Figure S5. (a) XPS Au 4f signal of a bare Au substrate (grey), and functionalized Au electrode with Zn-DPP molecule. (b) XPS Zn 2f signal of the functionalized Au electrode with Zn-DPP molecule.

Control blinking experiments also shows that only when the three components (the two pyridinyl and the Porphyrin) are present a molecular electrical connection (bridge) can be established.

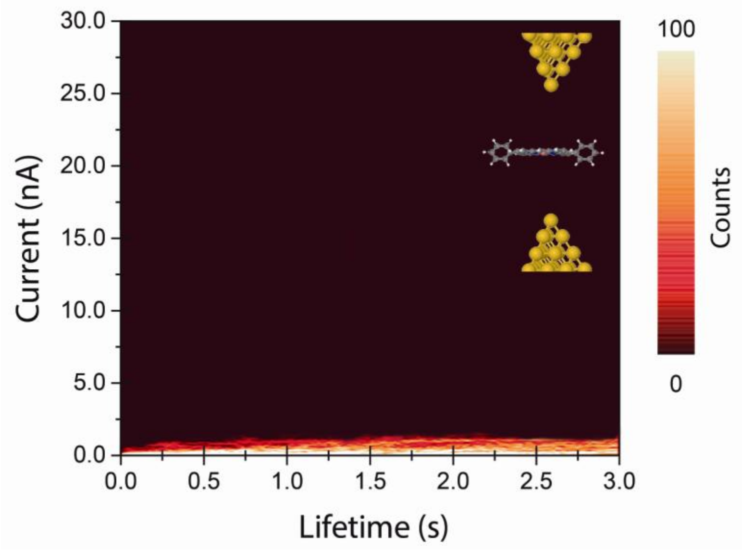

Figure S6. 2D blinking map in the absence of the pyridinyl units at the tip and substrate electrodes surfaces when porphyrin molecules are present in solution. All traces were set into a common time origin and baseline.

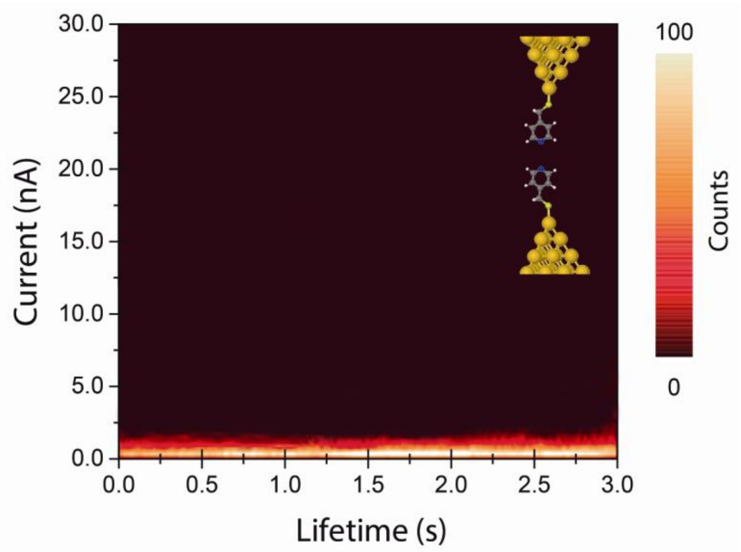

Figure S7. 2D blinking map in absence of the porphyrin molecules with both tip and surface functionalized with pyridinyl groups. All traces were set into a common time origin and baseline.

Supplementary references

S1. Xu, B.; Tao, N. J. Measurement of Single-Molecule Resistance by Repeated Formation of Molecular Junctions. Science 2003, 301 (5637), 1221–1223.

S2. Haiss, W.; Nichols, R. J.; van Zalinge, H.; Higgins, S. J.; Bethell, D.; Schiffrin, D. J. Phys. Chem. Chem. Phys. 2004, 6, 4330−4337

S3. Aragonès, A. C.; Haworth, N. L.; Darwish, N.; Ciampi, S.; Bloomfield, N. J.; Wallace, G. G.; Diez-Perez, I.; Coote, M. L. Electrostatic Catalysis of a Diels–Alder Reaction. Nature 2016, 531 (7592), 88–91
